# Osteocytes and Paget’s Disease of Bone

**DOI:** 10.1007/s11914-024-00863-5

**Published:** 2024-03-08

**Authors:** Hirofumi Tenshin, Jesus Delgado-Calle, Jolene J. Windle, G. David Roodman, John M. Chirgwin, Noriyoshi Kurihara

**Affiliations:** 1 Division of Hematology and Oncology, Department of Medicine, Indiana University, Indianapolis, IN USA; 2 Department of Physiology and Cell Biology, Winthrop P. Rockefeller Cancer Institute, University of Arkansas for Medical Sciences, Little Rock, AR USA; 3 Department of Human and Molecular Genetics, Virginia Commonwealth University, Richmond, VA USA; 4 Research Service, Roudebush Veterans Administration Medical Center, Indianapolis, IN USA

**Keywords:** Osteocyte, Osteoclast, Paget’s bone disease, Senescence, RANKL

## Abstract

**Purpose of Review:**

To describe the contributions of osteocytes to the lesions in Paget’s disease, which are characterized by locally overactive bone resorption and formation.

**Recent Findings:**

Osteocytes, the most abundant cells in bone, are altered in Paget’s disease lesions, displaying increased size, decreased canalicular length, incomplete differentiation, and less sclerostin expression compared to controls in both patients and mouse models. Pagetic lesions show increased senescent osteocytes that express RANK ligand, which drives osteoclastic bone resorption. Abnormal osteoclasts in Paget’s disease secrete abundant IGF1, which enhances osteocyte senescence, contributing to lesion formation.

**Summary:**

Recent data suggest that osteocytes contribute to lesion formation in Paget’s disease by responding to high local IGF1 released from abnormal osteoclasts. Here we describe the characteristics of osteocytes in Paget’s disease and their role in bone lesion formation based on recent results with mouse models and supported by patient data.

## Introduction

Sir James Paget, surgeon to Queen Victoria, described a disease of bone in 1877 and an unrelated disease of the breast earlier. Both disorders still bear his name. Paget’s disease of bone is characterized by localized areas of increased bone resorption coupled with exuberant new bone formation and with high circulating alkaline phosphatase activity. Paget’s disease occurs mostly in the elderly. The lesions are focal and solitary and do not spread. Both genetic and viral etiologies contribute to the phenotype of what is now known to be a spectrum of bone disorders [1•, 2].

The primary visible cellular abnormality in Paget’s disease resides in the osteoclasts, which are increased in size and multinucleation compared to normal osteoclasts. They secrete more interleukin-6 (IL-6) and insulin-like growth factor 1 (IGF1) and are hyperresponsive to 1,25-dihydroxyvitamin D_3_ [3–5]. The osteoclast as the primary site of cellular abnormality is supported by the efficacy of treatment with bisphosphonate inhibitors. A single infusion of zoledronic acid results in multi-year elimination of pagetic lesions, often for the life of the patient. Infrequent recurrences are at the original site [6]. Since osteoclasts arise from precursors that circulate in peripheral blood, how do pagetic lesions remain focal? This conundrum suggests that pagetic bone differs from uninvolved skeletal sites in the same patient and that the differences maintain the local persistence of the lesion. Osteocytes are immobile, long-lived, regulate osteoblast and osteoclast functions, and are subject to epigenetic reprogramming [7•], making them prime candidates to be central regulators of pagetic lesions.

**Fig. 1:**
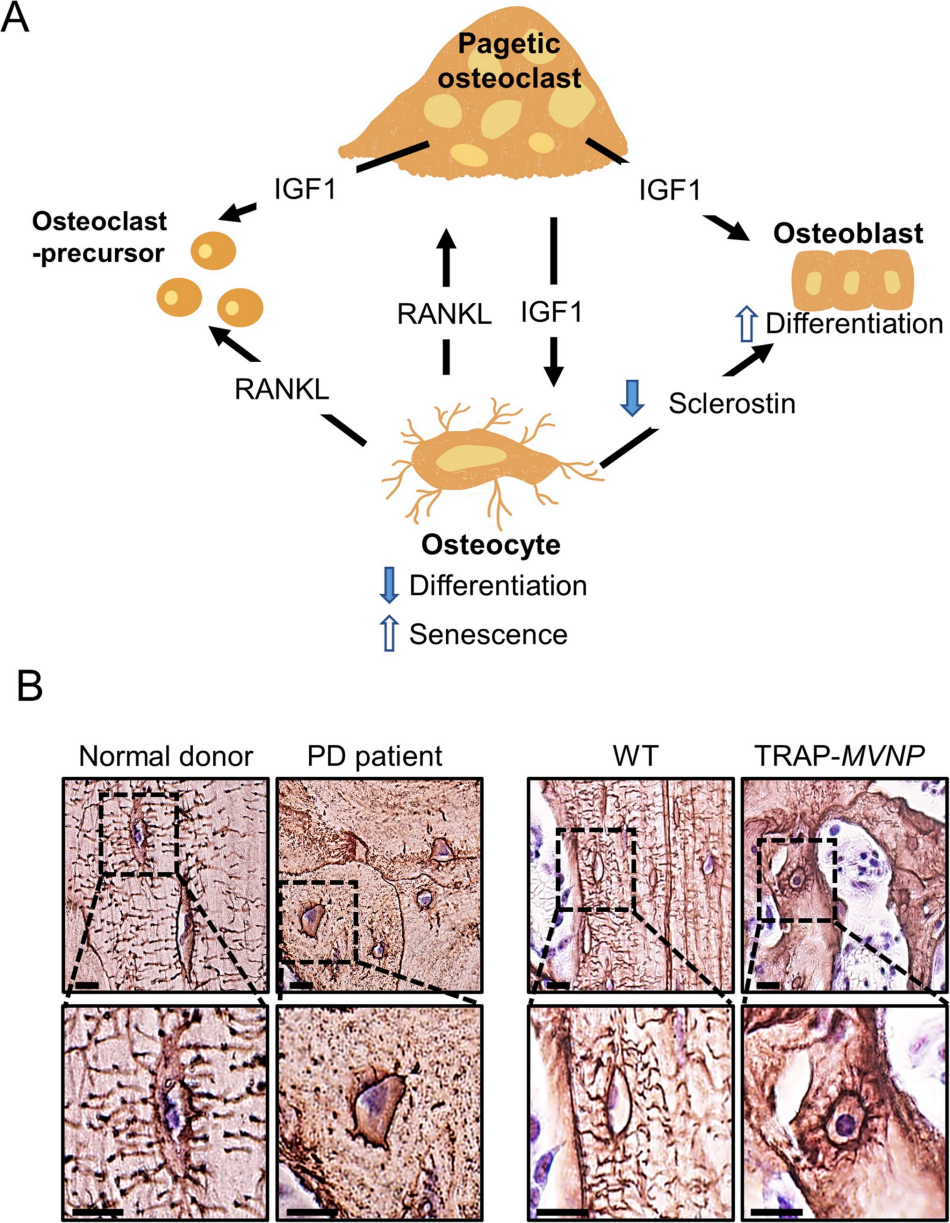
**A** Proposed role of osteocytes in pagetic bone lesions. Osteoclast-IGF1 induces osteocyte senescence and RANKL production, suppresses osteocyte sclerostin, and enhances the formation of pagetic osteoclasts, leading to increased local bone destruction coupled with disorganized new bone formation (from [23••]). **B** Images of osteocytes from a Paget’s disease patient and a TRAP-*MVNP* mouse. Spinal bone sections were treated with Ploton silver to stain the canalicular and cement lines. The Paget’s patient was a 59-year-old male, and the normal subject was a 58-year-old male; both bone samples were from the spine (provided by Dr. Brendan Boyce, University of Rochester). TRAP-*MVNP* and WT mouse sections were taken 500 µm below the distal growth plate of 20-month-old femurs. Scale bars are 10 µm. Images are of different sections from the samples published in Miyagawa et al. [23••]

We hypothesize that osteocytes play a key role in pagetic bone lesions. Figure 1A cartoons how paracrine interactions between pagetic osteoclasts and adjacent osteocytes might sustain the abnormal phenotype of pagetic bone. Pagetic osteoclasts secrete IGF1, which increases senescence of nearby osteocytes; these cells in turn secrete more receptor activator of nuclear factor kappa beta ligand (RANKL) and less sclerostin: the former resulting in more osteoclastic osteolysis, and the latter permitting more osteoblastic new bone formation. In what follows, we review the genetic and environmental conditions that can contribute to Paget’s disease and then focus on experiments with transgenic mice where measles virus nucleocapsid protein (MVNP) is targeted to the osteoclast lineage. Around 1 year of age, these mice develop bone lesions that resemble those seen in patients with Paget’s disease.

Both genetic and environmental factors can cause abnormal osteoclast activity in Paget’s disease, recently reviewed by Gennari et al. [8•]. Genes where mutations trigger Paget’s disease lesions include sequestosome 1 (p62), ZNF687, and profilin1. Mutations in other genes, such as optineurin and RANK, predispose to the development of pagetic bone lesions but are insufficient to cause them. Triggering mutations and environmental factors have been confirmed in mouse models, although Paget’s disease lesions are usually found in mice at >1 year of age, making such animal experiments slow and expensive. As is characteristic of the human disease, Paget’s disease in mice is responsive to treatment with bisphosphonate anti-resorptive agents [9].

Over 21 mutations in human *SQSTM1/p62* (a scaffold protein with a key role in autophagy) are linked to familial Paget’s disease (about 30% of all Paget’s disease cases), with *p62*^*P392L*^ the most frequent [10•, 11, 12]. We found that osteoclast precursors expressing *p62*^*P392L*^ did not form bone lesions and lacked the osteoclastic characteristics of Paget’s disease [10•]. Knock-in mice carrying the murine equivalent of human *p62*^*P392L*^ (*p62*KI mice) had increased osteoclast precursors but histologically normal bones [13]. Daroszewska and co-workers [11] reported bone lesions in a similar *p62*KI mouse, but these were atypical of Paget’s disease. The results suggest that additional factors contribute to the development of Paget’s disease due to *p62* mutation [14]. Pagetic bone lesions also occur in a variety of complex genetic disorders, such as those involving profilin 1 and valosin-containing protein/p97 [15, 16], but these multisystem proteinopathies are early onset and have extra-skeletal symptoms along with multiple bone lesions, unlike the late onset, solitary presentation of common Paget’s disease [17].

Environmental factors, including infections by paramyxoviruses (e.g., measles virus or canine distemper virus), have been implicated in the pathogenesis of Paget’s disease [1•] since 1976, when Miller and Singer described viral nuclear inclusions in pagetic osteoclasts [8•]. Despite the course of nearly 50 years and more than 100 publications on measles virus in Paget’s disease, a viral contribution to clinical Paget’s disease remains controversial. Some groups have detected viral inclusions and paramyxoviral transcripts and antigens in the osteoclasts of pagetic lesions [18, 19], while others have exhaustively failed to do so [20]. Part of the challenge is explaining how abnormalities in osteoclast morphology and function could appear in an elderly patient with Paget’s disease decades after a viral infection that is no longer detectable.

Precursor cells differentiate into pagetic osteoclasts in vitro when manipulated to express *MVNP* protein (which in measles-infected cells has transcriptional regulatory activity) [12, 21••]. When an *MVNP* transgene was targeted to the osteoclast using the tartrate-resistant acid phosphatase (TRAP) promoter, 43% of 16–20-month-old mice developed bone lesions characteristic of Paget’s disease, with abnormal osteoclasts and irregular, jigsaw-puzzle shaped bone particles that are hallmarks of Paget’s disease [21••, 22, 23••]. Interestingly, marrow cultures of osteoclasts from Paget’s patients carrying the *p62*^*P392L*^ mutation formed pagetic osteoclasts in vitro only if they co-expressed *MVNP*. Pagetic differentiation was blocked by antisense RNA to *MVNP* [10•]. When *p62*KI mice were bred to TRAP-*MVNP* mice, the *p62*KI/*MVNP* mice developed greater numbers of pagetic osteoclasts with age and bone lesions that were strikingly like those seen in patients [10•]. *MVNP*-transgenic osteoclasts secrete abundant IGF1 and interleukin-6 (IL-6) [14]. In *MVNP* mice, deletion of IL-6 [5] or conditional knockout of IGF1 in the osteoclast lineage abrogated formation of pagetic osteoclasts and bone lesions in vivo [22].

## Roles of IL-6 and IGF1 in Paget’s Disease

Paget’s disease patients have elevated IL-6 in marrow plasma and peripheral blood [3], and their osteoclasts express high IL-6 [24]. *MVNP* induces high IL-6 expression via downregulation of FoxO3/Sirt1 signaling, increasing the formation of pagetic osteoclasts and bone lesions [24]. IL-6 ablation in *MVNP* mice blocked development of pagetic osteoclasts and bone lesions in vivo [5]. However, transgenic overexpression of IL-6 in osteoclasts did not induce a pagetic phenotype in osteoclasts or pagetic lesions in mice [5], suggesting that other factors induced by *MVNP* are needed for the development of Paget’s disease. IL-6 can stimulate the differentiation of primary calvarial osteoblasts and induce RANKL in osteoblastic precursors [25], but its overexpression in mice reduces osteoblast numbers, and it may inhibit bone formation in vivo. Wu et al. reported [26] that IL-6 enhanced osteocyte-mediated osteoclast formation by increasing RANKL. Thus, increased IL-6 from pagetic osteoclasts could enhance RANKL production by osteocytes to further increase local osteoclast formation. Immunohistochemical studies of bones from Paget’s disease patients showed that pagetic osteoclasts contained abundant platelet-derived growth factor, transforming growth factor-β and IGF1, as well as IL-6 [27]. Gene expression profiling studies of highly purified cells from *MVNP*, *p62*KI, and wild type (WT) mice showed that *MVNP*-expressing osteoclasts contained large amounts of IGF1 mRNA, while osteoclasts from WT and *p62*KI mice did not [14]. Importantly, conditional knockout of IGF1 in osteoclasts blocked the development of pagetic bone lesions and restricted osteocyte differentiation in *MVNP* mice [22].

## Characteristics of Osteocytes in Paget’s Disease

Osteoclasts are hyper-multinucleated and increased in number in pagetic bone lesions [10•, 28]. The lesions are solitary and persist for years, while active osteoclasts are short-lived derivatives from circulating precursors. The persistent memory component of Paget’s lesions could be provided by long-lived osteocytes resident within bone [29]. Unusual canalicular bone resorption around osteocytes in Paget’s disease patients was reported as early as 1968 [30], but this is not unique to Paget’s disease. Ultrastructural changes were later reported in pagetic osteocytes [31]. Viral inclusions and sequences have been detected repeatedly in pagetic osteoclasts, but paramyxoviral transcripts without viral inclusions have been found only infrequently in osteoblasts and osteocytes. The photomicrographs in Fig. 1B, like previously published images [21••, 22, 23••], show Ploton silver staining of bones from a Paget’s disease patient, an *MVNP* mouse, a normal donor, and a WT mouse. The canalicular lengths of osteocytes in *MVNP* mice were shorter than in WT mice but did not differ between sexes [23••].

The osteocytes in a bone biopsy from a patient with Paget’s disease showed reduced sclerostin staining compared to a control bone biopsy from a normal patient [23••]. Sclerostin expression and dendritic processes of the osteocytes in *MVNP* mice were significantly reduced, compared to WT mice, in regions of bone that lacked pagetic lesions. Osteocyte sclerostin expression and dendritic processes were further reduced in *MVNP* mice at sites of pagetic bone lesions, which also had lower numbers of sclerostin-expressing osteocytes per bone area and decreased canalicular length compared with *MVNP* mice without pagetic bone lesions or WT mice. Circulating serum sclerostin concentrations were similar in all WT and *MVN*P mice [23••], perhaps because the osteocytes in the pagetic bone lesions are a very low percentage of all the sclerostin-secreting osteocytes in the mice. At least five papers report circulating levels of sclerostin in Paget’s disease patients, with two suggesting it is increased, while three found no change [32–34].

Osteocyte differentiation is an active process [35]. These cells develop from a polygonal osteoblastic precursor to one with cytoplasmic extensions directed toward the mineralizing front and that can extend to the bone surface to interact with other cells, bone marrow cells, and the vascular space [36]. Osteocyte differentiation occurs as precursors become embedded in osteoid and begin to extend dendritic processes, followed by expression of DMP1 and CapG, proteins which regulate the cytoskeleton. Mature osteocytes within mineralized bone secrete sclerostin, FGF23, and ORP150, a factor thought to protect the cells from hypoxia [37, 38].

Sclerostin secretion and dendrite formation are characteristics of mature osteocytes [39], which can also make IGF1 [7•], while IGF1 enhances osteocyte differentiation stimulated by parathyroid hormone [40]. We analyzed mRNAs in primary osteocytes isolated by collagenase digestion of long bones from 20-month-old WT and *MVNP* mice. Sclerostin (*Sost*) mRNA in cells from *MVNP* mice was reduced by 30% compared to WT, while *Igf1* mRNA was unchanged [23••]. Fluorescent immunostaining of osteocytes from *MVNP* mice showed decreased average intensity of staining for the maturation markers DMP1 and sclerostin, compared to WT mice [23••].

Only a limited number of osteocytes can be obtained by collagenase digestion of mouse bones, so we studied osteoblasts and osteocytes from cells that grow out of bones in tissue culture after removal from WT and *MVNP* mice [23••]. Future experiments would assess local sclerostin expression by osteocytes within histological sections from pagetic patients’ bone lesions compared to controls to determine if osteocyte maturation is impaired or delayed in Paget’s disease.

## Senescence and Osteocytes

Farr and colleagues recently characterized senescent osteocytes using highly purified cells from 6- and 24-month-old mice. Old mice showed a 6-fold increase in senescent osteocytes (11% vs. 2%), which displayed a senescence-associated secretory phenotype with high expression of 23 genes, including IL-6, IL-1, NF-κB, GM-CSF, IL-8, MCP1, and M-CSF, a gene signature with pro-osteoclastogenic potential [41••]. Upregulation of p16^INK4A^, telomere shortening, and decondensation of pericentromeric satellite DNA marked the senescent cells. Tran et al. [42] reported that prolonged exposure of mouse and human fibroblasts to IGF1 induced premature cellular senescence. IGF1 inhibited SIRT1 deacetylase activity, resulting in increased p53 acetylation, stabilization, and activation, leading to premature senescence. We hypothesize that a local subset of osteoclasts in Paget’s disease patients may undergo senescence due to exposure to high local levels of osteoclast-IGF1, forming a niche that activates precursors to increase pagetic-OCL formation. Mutations in *p62* could enhance pagetic osteoclast activation of senescent osteocytes through increased NF-κB signaling [43].

## Senescent Osteocytes are Important Sources of RANKL in Paget’s Disease

Senescent osteocytes express RANKL, which contributes to the cortical bone loss that occurs with age [44••]. Yan and colleagues recently identified an intronic enhancer of RANKL that may be selectively activated in osteocytic cells and could be stimulated by senescence [45•], suggesting a molecular basis for RANKL regulation in osteocytes, with therapeutic implications for various skeletal disorders.

The regulation of osteocyte RANKL production during senescence [43, 44••, 45•] was examined by comparing p16^INK4A^ and RANKL in *MVNP* and WT mice by immunofluorescent staining of single cells. Both p16^INK4A^ and RANKL were more highly expressed in *MVNP* than in WT mice (preliminary data, not shown). p16^INK4A^- and RANKL-expressing osteocytes in *MVNP* mice were increased 2.5- to 3.5-fold compared with WT. Moreover, p16^INK4A^/RANKL double-positive osteocytes accounted for 25% of total osteocytes in *MVNP* mice, 3-fold higher than in WT [23••]. The results confirm previous observations that osteocyte RANKL production is part of a senescence-associated secretory phenotype. Twenty-five percent of osteocytes in pagetic bone lesions showed senescence markers and secreted RANKL [23••]. These phenotypic changes in osteocytes may be regulated by IGF1 signaling, which is known to induce intracellular oxidative burden and associated oxidative damage and can lead to premature senescence. Osteoclast-secreted IGF1 in Paget’s disease could thus act on nearby osteocytes to induce senescence and increase local RANKL.

## Conclusions

Paget’s disease of bone is characterized by skeletal sites of persistent high bone formation coupled to high bone resorption with a primary abnormality of osteoclasts. Such lesions do not spread, and the osteocytes in them are now known to be abnormal both morphologically and biochemically. They display altered canalicular processes, express increased RANKL, which may contribute to increased osteolysis, and secrete less sclerostin, which could permit increased new bone formation. High local concentrations of IGF1 secreted from pagetic osteoclasts increase markers of osteocyte senescence; secretion of RANKL is induced from senescent osteocytes and contributes to the maintenance of pagetic bone lesions. It is presently uncertain whether osteocytes also play a major role in the coupling between osteoclastic bone destruction and osteoblastic new bone formation characteristic of Paget’s disease of bone.

## Future Directions

The local elevation of IGF1 in pagetic lesions appears to act by induction of osteocyte senescence. It is currently unknown whether elevated osteoclast-IGF1 and osteocyte senescence are central factors in the development of pagetic lesions in Paget’s disease due to causes such as mutations in p62 or non-measles environmental factors. In future studies, pagetic bone samples from patients with Paget’s disease and from mouse models could be stained for osteocyte morphology and expression of RANKL, sclerostin and senescence markers and for IGF1 in osteoclasts.

Bone formation remains coupled to resorption in pagetic lesions. This is in part due to the expression of the coupling factors EphB2 on osteoclasts and EphB4 on osteoblasts, but may also be regulated by osteocytes, making this another area for future study. It remains unclear what initiates pagetic lesions. Since Paget’s disease occurs focally and late in life, it could be the result of a stochastic event at the site of a future lesion, perhaps involving a local cluster of senescent osteocytes which attract osteoclast precursors. This question could be addressed by homing experiments with transgenic mice and genetically marked osteoclast precursor cells.

Paget’s disease of bone is efficiently treated with anti-resorptive agents. Bisphosphonates not only kill osteoclasts but increase osteocyte viability, which could contribute to their efficacy against Paget’s, offering another area for future study. The altered phenotype of osteocytes within persistent pagetic lesions suggests that the cells have altered gene expression that could be due to epigenetic reprogramming, yet another area for future investigation. Although now an easily treated skeletal disorder, Paget’s disease of bone continues to provide insights into the cellular regulation of bone turnover and to suggest new roles for osteocytes as central controllers of osteoclast and osteoblast actions and interactions.

## Data Availability

Not applicable.
